# Charting Cervical Spinal Cord Morphometry Across the Lifespan

**DOI:** 10.64898/2026.06.03.729823

**Published:** 2026-06-08

**Authors:** Kurt G. Schilling, Michael E. Kim, Matthew Amandola, Chenyu Gao, Karthik Ramadass, Praitayini Kanakaraj, Sam Bogdanov, Gaurav Rudravaram, Nancy R. Newlin, Derek Archer, Timothy J. Hohman, Angela L. Jefferson, Victoria L. Morgan, Alexandra Roche, Dario J. Englot, Murat Bilgel, Lori L. Beason Held, Luigi Ferrucci, Laurie Cutting, Laura A. Barquero, Micah A. D’archangel, Tin Q. Nguyen, Kathryn L. Humphreys, Yanbin Niu, Sophia Vinci-Booher, Carissa J. Cascio, The HABS-HD Study Team, Alzheimer’s Disease Neuroimaging Initiative, The BIOCARD Study Team, Zhiyuan Li, Daniel Moyer, Simon N. Vandekar, Panpan Zhang, Samuelle St-Onge, Sandrine Bédard, Jan Valošek, Benjamin De Leener, Julien Cohen-Adad, John C. Gore, Seth Smith, Bennett A. Landman

**Affiliations:** 1 Vanderbilt University Medical Center, Department of Radiology and Radiological Sciences, Nashville, TN, USA; 2 Vanderbilt University, Department of Computer Science, Nashville, TN, USA; 3 Vanderbilt University, Department of Electrical and Computer Engineering, Nashville, TN, USA; 4 Vanderbilt University, Medical Scientist Training Program, Nashville, TN, USA; 5 Vanderbilt University Medical Center, Vanderbilt Memory and Alzheimer’s Center, Nashville, TN, USA; 6 Vanderbilt University Medical Center, Vanderbilt Genetics Institute, Nashville, TN, USA; 7 Vanderbilt University, Vanderbilt Brain Institute, Nashville, TN, USA; 8 Vanderbilt Alzheimer’s Disease Research Center, Nashville, TN, USA; 9 Vanderbilt University Medical Center, Department of Medicine, Nashville, TN, USA; 10 Vanderbilt University Medical Center, Department of Neurology, Nashville, TN, USA; 11 Vanderbilt University, Department of Psychology and Human Development, Nashville, TN, USA; 12 Vanderbilt University Medical Center, Department of Psychiatry and Behavioral Sciences, Nashville, TN, USA; 13 Vanderbilt University Institute of Imaging Science, Nashville, TN, USA; 14 Vanderbilt University, Department of Biomedical Engineering, Nashville, TN, USA; 15 Vanderbilt University Medical Center, Department of Neurological Surgery, Nashville, TN, USA; 16 Laboratory of Behavioral Neuroscience, National Institute on Aging, National Institutes of Health, Baltimore, MD, USA; 17 Peabody College of Education and Human Development, Department of Special Education, Nashville, TN, USA; 18 University of Kansas, Life Span Institute and Department of Psychology, Lawrence, KS, USA; 19 (No affiliation data provided); 20 Department of Computer Science, Park University, Parkville, MO, USA; 21 Vanderbilt University Medical Center, Department of Biostatistics, Nashville, TN, USA; 22 NeuroPoly Lab, Institute of Biomedical Engineering, Polytechnique Montréal, Montréal, QC, Canada; 23 CHU Sainte-Justine Research Centre, Montréal, QC, Canada; 24 Spinal Cord Injury Center, Balgrist University Hospital, University of Zürich, Zürich, Switzerland; 25 Department of Neurosurgery, Faculty of Medicine and Dentistry, Palacký University Olomouc, Olomouc, Czechia; 26 Department of Biomedical Engineering, University Hospital Olomouc, Olomouc, Czechia; 27 Centre de recherche du CHU Sainte-Justine, Montréal, Canada; 28 Department of Computer Engineering and Software Engineering, Polytechnique Montréal, Montréal, Canada; 29 Functional Neuroimaging Unit, CRIUGM, Université de Montréal, Montreal, QC, Canada; 30 Mila - Quebec AI Institute, Montreal, QC, Canada

## Abstract

Spinal cord morphometry provides essential biomarkers of neurological health, but clinical interpretations are confounded by inter-subject variability and a lack of normative references across the full human lifespan. We address this gap by generating the first comprehensive lifespan charts for cervical spinal cord morphometry. We leveraged 30 population-based brain MRI datasets, aggregating 78,269 scans from 41,042 individuals (ages 0–100) whose imaging protocols included cervical cord coverage. To overcome contrast variability, we employed a state-of-the-art contrast-agnostic deep learning segmentation method, extracting cross-sectional area (CSA), anteroposterior (AP) and right–left (RL/transverse), and shape indices (compression ratio, eccentricity, and solidity) from C1 to C7. Normative trajectories were modeled using Generalized Additive Models for Location, Scale, and Shape (GAMLSS). The resulting charts reveal distinct non-linear lifespan changes: rapid growth through childhood and adolescence, peak maturation occurring in early-to-mid adulthood (e.g., mid-30s for CSA), followed by gradual decreases. Significant regional variations along the cervical cord and consistent sex differences (males > females for size metrics) were quantified. Spinal cord trajectories showed strong temporal coupling with brain white matter and brainstem volumes, suggesting integrated CNS development and aging. These lifespan charts provide a robust normative framework, enabling age- and sex-specific centile scoring of individual spinal cord morphometry. This resource offers a critical tool for differentiating typical variation from pathological changes, enhancing the clinical utility of spinal cord MRI in studies of development and neurodegeneration.

## Introduction

Spinal cord morphometry provides quantitative measures reflecting macrostructural integrity that are directly relevant to neuroscience and clinical care [1–4]. Cross-sectional area (CSA) and related geometric indices (anteroposterior and transverse diameters, compression ratio, eccentricity, solidity) are sensitive to cord tissue loss and shape changes across neurological conditions [5] - including multiple sclerosis [6, 7], amyotrophic lateral sclerosis [8–10], spinal cord injury [11], and degenerative cervical myelopathy [5, 12] - and can serve as biomarkers of disease monitoring and prognosis. In multiple sclerosis, spinal cord atrophy is detectable from the earliest stages and correlates with motor disability [13–15]; in degenerative cervical myelopathy, automated morphometric profiling complements radiological assessment of stenosis and helps standardize detection of compressed cord [5, 12, 16]. To interpret these measurements and disentangle pathology from natural inter-subject variation, a lifespan reference for normative values is needed.

Despite this clinical potential, broad adoption is limited by measurement variability that complicates subject-specific interpretation and reduces statistical power in group studies. A century of reports - from post-mortem measurements [17, 18] to modern MRI [19–21] - has yielded disparate “normative” values for spinal cord diameters and area (for a review, see [3]), with several factors driving this inconsistency. First, biological sources such as age, sex, and individual anatomical differences produce between-person dispersion [1, 22–25]. Second, methodological sources introduce bias across studies, including scanner vendor, acquisition contrast/protocol, and analysis software assumptions about cord boundaries [26–28]. Finally, variability arises from spatial localization, encompassing both the precision of identifying anatomical landmarks and the inherent neuroanatomical variance between vertebral levels and functional spinal cord segments [1]. Together these effects hinder cross-site aggregation, meta-analysis, and reliable longitudinal tracking.

Normative modeling addresses these challenges by adapting the logic of pediatric growth charts [29, 30] to neuroimaging. Recent brain-wide “charts” provide age- and sex-stratified trajectories and variance across the lifespan [31, 32]. Crucially, such models estimate the full population-level distribution of a given biological measure - not just the mean - so individual measurements can be expressed as centile scores relative to age-appropriate expectations, enabling standardized detection of atypical development or atrophy [31].

The main barrier to creating comprehensive spinal cord charts has been the lack of large-scale, dedicated imaging datasets spanning the full human lifespan. While valuable prior work laid the foundation by creating normative databases for specific populations, such as the PAM50 spinal cord template for adults [20, 21, 33], and by exploring normalization strategies to reduce inter-subject variability in adult and pediatric cohorts [1, 5, 12, 23, 34], these efforts were often limited by narrow age ranges, modest sample sizes, a focus on single vertebral levels, or simplified models unable to capture age-varying variance. Here, we overcome these constraints by leveraging numerous large-scale, population-based brain neuroimaging datasets whose imaging protocols included cervical cord coverage. The feasibility and reliability of using such data for upper cervical cord morphometry are well-established [35, 36], presenting an exemplary opportunity to utilize existing data resources. However, aggregating data across these diverse cohorts also introduces significant methodological challenges, primarily MRI contrast variability (across sites or contrasts). To address this, we employ a recently developed, state-of-the-art, contrast-agnostic segmentation method [2, 37], which provides consistent morphometric estimates across different scanners, sites, and protocols.

Our goal is to create vertebral-level, sex-stratified normative charts of cervical spinal cord morphometry from birth to 100 years. Using a dataset of 78,269 scans from 41,042 individuals across 30 population-based neuroimaging studies, we (i) model the non-linear trajectories of spinal cord growth, maturation, and atrophy; (ii) identify key lifespan milestones (e.g. age of peak CSA and diameters); (iii) quantify annualized rates of change for clinical benchmarking across life stages; (iv) characterize population variability; (v) delineate sex-specific trajectories; and (vi) relate cord morphometry to brain volumes across development and aging.

## Materials and Methods

This study involved three main stages: (i) aggregation and curation of a large-scale, multi-site neuroimaging dataset; (ii) automated processing of all images to extract spinal cord and brain morphometric features; and (iii) normative modeling of these features to generate lifespan charts.

### Study Cohorts

**Figure 1.**
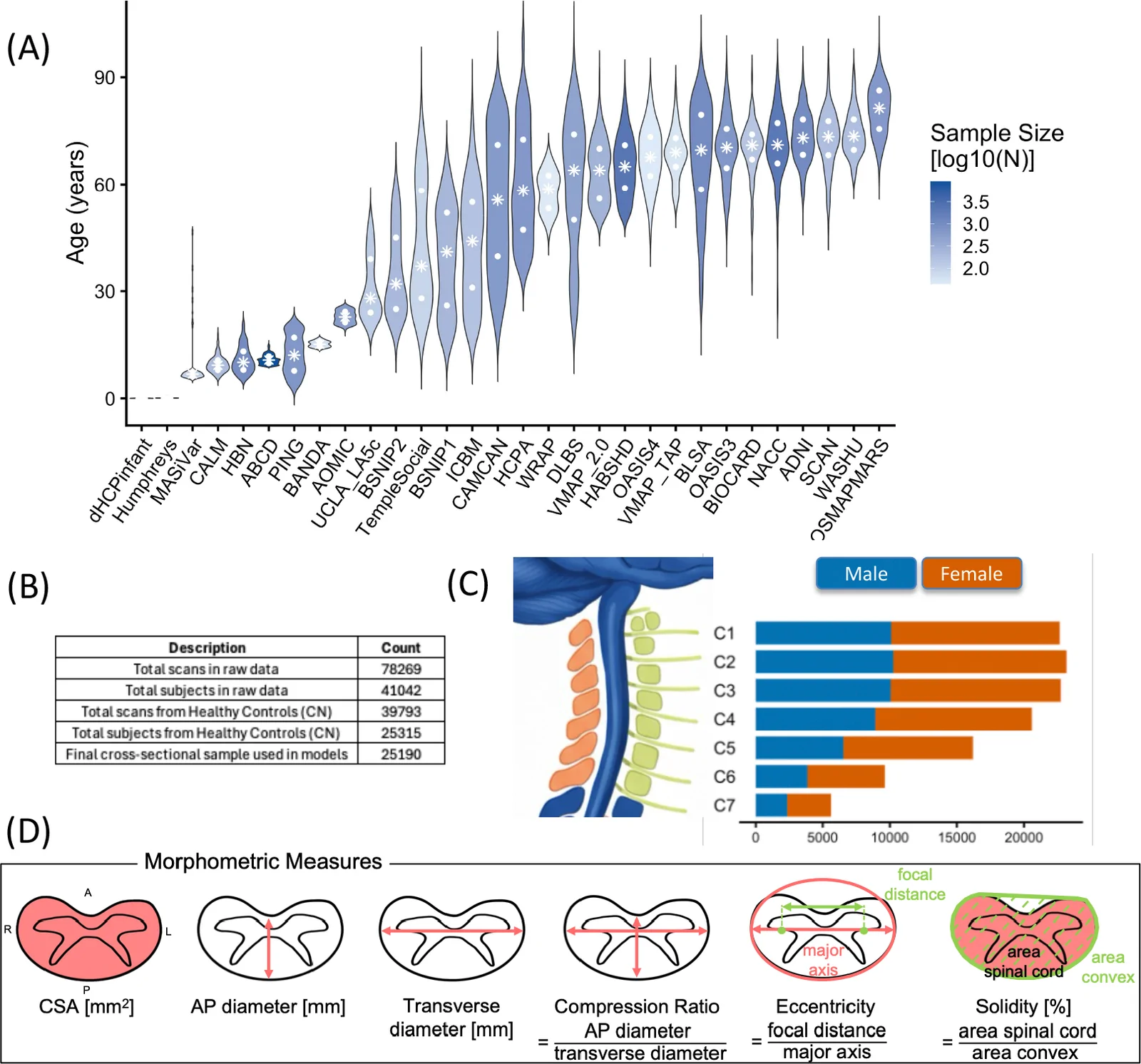
Overview of Study Cohorts and Extracted Spinal Cord Morphometry. We analyzed data from 30 population-based neuroimaging datasets, totaling 78,269 imaging sessions from 41,042 individuals (ages 0–100) whose brain MRI protocols incidentally included cervical cord coverage (A). After excluding scans from individuals with clinical diagnoses and selecting a single scan from subjects with longitudinal data, a final cross-sectional cohort of 25,190 healthy subjects was established for normative modeling (B). Due to variable field-of-view across studies, the sample size available for analysis varied by vertebral level (C). From the resulting spinal cord segmentations, six primary morphometric features were extracted at each level for analysis: Cross-Sectional Area (CSA), Anterior-Posterior (AP) diameter, Right-Left (RL) diameter, Compression Ratio, Eccentricity, and Solidity (D) (cord illustration recreated from [21]).

This work builds upon prior large-scale data aggregation efforts which collated imaging data from numerous publicly available, population-based neuroimaging studies [38]. From this initial collection, we identified 30 primary studies whose structural T1-weighted MRI acquisitions included incidental coverage of the cervical spinal cord. This resulted in a comprehensive dataset of 78,269 imaging sessions from 41,042 unique individuals spanning ages 0–100 years (Figure 1A). Additional information on datasets, sample sizes, and age ranges are provided in **Supplementary Table 1**.

To construct a normative cohort, we first applied inclusion criteria to retain only scans only from participants designated as healthy or cognitively normal controls (as identified from each primary neuroimaging cohort/consortium). From this healthy subset, which included individuals with both cross-sectional and longitudinal data (N=39,793 imaging sessions from 25,315 subjects), we created a final cross-sectional modeling dataset by randomly selecting a single scan from each participant with multiple time points, a strategy consistent with large-scale charting initiatives [31]. This procedure yielded a final cohort of 25,190 healthy individuals used for generating the normative lifespan charts (Figure 1B). We retained metadata (dataset identifier, subject/session, age, sex) for downstream robustness analyses (e.g. leave-one-site-out, bootstrap stability).

Due to inherent differences in the imaging field-of-view across the aggregated studies, the number of subjects with available data varied across the cervical spine. The sample size was largest at the upper cervical levels (e.g., C1-C3) and progressively decreased at more caudal levels. This resulted in a vertebral-level-specific sample size for our normative models, as illustrated in Figure 1C.

### Image processing

#### Spinal cord morphometry

All T1-weighted images were processed with the Spinal Cord Toolbox (SCT, v7.1) [39]. To handle variability in image contrast across the aggregated datasets, we used a state-of-the-art, contrast-agnostic deep learning model to automatically segment the spinal cord from all T1-weighted images (*sct_deepseg spinalcord*) [2]. This method offers a key advantage critical for this large-scale study: it provides robust and consistent segmentation performance regardless of the input image contrast (i.e., differences in acquisition parameters across sites). Vertebral levels were extracted using a tool for automatic instance segmentation of all vertebrae (*sct_deepseg totalspineseg*), that is also robust to various MRI contrasts, acquisition orientations, and resolutions.

Following segmentation, a suite of morphometric features was extracted at each vertebral level in the subject native space using (*sct_process_segmentation*) The primary features analyzed in this study include (Figure 1D):

- Cross-Sectional Area (CSA) (mm^2^): The total area of the spinal cord in the axial plane (on a centerline-orthogonal axial slice), calculated from the binary segmentation by summing the voxel-wise probabilities. CSA is a primary indicator of spinal cord atrophy.
- Anterior-Posterior (AP) Diameter (mm): The maximum diameter of the spinal cord along the anterior-posterior axis. Calculated as the AP axis of a fitted ellipse.
- Right-Left (RL) / Transverse Diameter (mm): The maximum diameter of the spinal cord along the right-left axis, also referred to as the transverse diameter. Calculated as the RL axis of a fitted ellipse.
- Compression Ratio (a.u.): The ratio between the AP and RL diameters, which provides a measure of cord flattening (lower value indicates greater flattening).
- Eccentricity (a.u.): A measure of the spinal cord’s deviation from a perfect circle, calculated from an ellipse with the same second-order moments as the cord segmentation (range 0–1; values nearer 0 indicate a more circular cross-section).
- Solidity: The ratio of the CSA to the area of its convex hull, which quantifies the convexity of the cord shape and is sensitive to local indentations (lower values indicate greater indentation/non-convexity)

#### Brain volumetrics

To investigate the relationship between brain and spinal cord morphometry, we processed all T1-weighted images using FreeSurfer (*recon-all*, v7.2.0) [40]. This widely used pipeline performs automated cortical reconstruction and subcortical segmentation, providing reliable volumetric measurements of various brain structures. For comparative analysis with spinal cord metrics, we extracted the following primary brain volumes: Total Brain Volume, Total Gray Matter Volume, Total White Matter Volume, Thalamus Volume (right + left), and Brainstem Volume.

### Normative modeling and lifespan charting

We modeled age-dependent normative trajectories using Generalized Additive Models for Location, Scale, and Shape (GAMLSS) [41] (as implemented in R as the gamlss package), following the framework adopted for pediatric growth [42] and recent large-scale brain charts [31, 43]. Each outcome (per-level cord morphometric or global brain volume) was fit with the Generalized Gamma (GG) family, with the location (μ) and scale (σ) parameters expressed as smooth, non-linear functions of age and sex. Age effects were modeled with fractional polynomials (order 2 for μ; order 1 for σ), and sex entered both location and scale as a fixed effect. To account for between-study/site heterogeneity, we included dataset-level random effects in both μ and σ, yielding site-robust population estimates while retaining within-dataset information. In the GG family, the shape parameter (ν) - which governs distributional skewness - was treated as constant (i.e., not modeled as a function of covariates) in our fits. Model adequacy was assessed independently using standard GAMLSS diagnostics, including worm plots and empirical-vs-model centile checks, to verify that fitted curves reproduced observed trends.

Outlier handling prior to fitting used two safeguards: (i) dataset- and level-specific screening for implausible values suggestive of segmentation or coverage error, and (ii) exclusion of observations exceeding ±3 SD relative to the dataset-level mean for that feature and level. Models were fit on the healthy cross-sectional cohort, separately for each vertebral level (C1–C7) and morphometric feature.

The output was a set of continuous, age- and sex-specific centile curves that constitute the spinal-cord lifespan charts, describing the population distribution via μ, σ (and constant ν). For visualization we show canonical centiles (2nd, 5th, 10th, 25th, 50th, 75th, 90th, 95th, 98th). From the fitted median (50th-centile) curve we derive two summaries used throughout the Results: the annualized rate of change (from the first derivative; also expressed as %/year) and the age of peak (maturation; derivative zero-crossing with negative curvature). For interpretability, rates are summarized across predefined lifespan stages: Infancy (0–1 y), Early Childhood (1–6 y), Late Childhood (6–12 y), Adolescence (12–20 y), Young Adulthood (20–40 y), Middle Adulthood (40–60 y), and Late Adulthood (60–100 y).

## Results

### Non-linear Dynamics of Spinal Cord Morphometry Across the Lifespan

**Figure 2.**
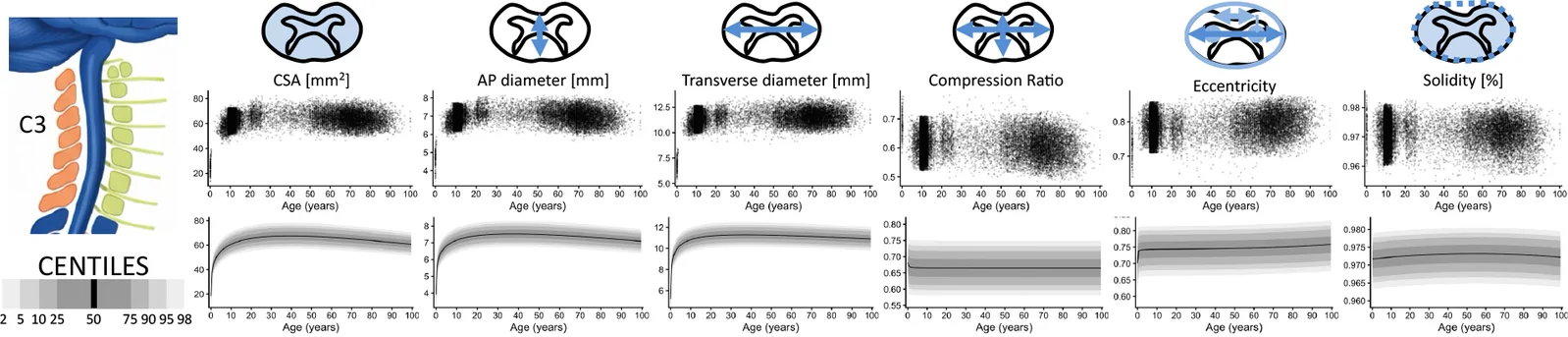
Cervical Spinal Cord Growth Charts Across the Lifespan. Lifespan trajectories for six morphometric features are shown at the representative C3 vertebral level. Individual data points from the healthy cohort (n>25,000) are plotted, along with the estimated centile curves (2nd, 10th, 25th, 50th, 75th, 90th, 98th) derived from GAMLSS models. Measures of cord size, including Cross-Sectional Area (CSA), Anterior-Posterior (AP) Diameter, and Right-Left (RL) Diameter, follow a characteristic non-linear trajectory of rapid growth through childhood, a plateau in young adulthood, and a gradual decrease in later life. In contrast, measures of cord shape exhibit distinct lifespan dynamics: Compression Ratio shows a subtle decline with age, Eccentricity demonstrates a gradual increase, and Solidity remains largely stable before a slight decrease in late adulthood.

We generated normative lifespan charts for cervical spinal cord morphometry by applying GAMLSS to our large-scale, cross-sectional cohort. At the representative C3 level (Figure 2), the raw data and fitted centiles show nonlinear trajectories across the lifespan. CSA and AP/RL (transverse) diameters rise rapidly through childhood and adolescence, plateau in young adulthood, and decreases in later life. Other shape metrics follow different patterns: compression ratio generally decreases with age, eccentricity increases modestly, and solidity is largely stable with a slight late-life reduction.

**Figure 3.**
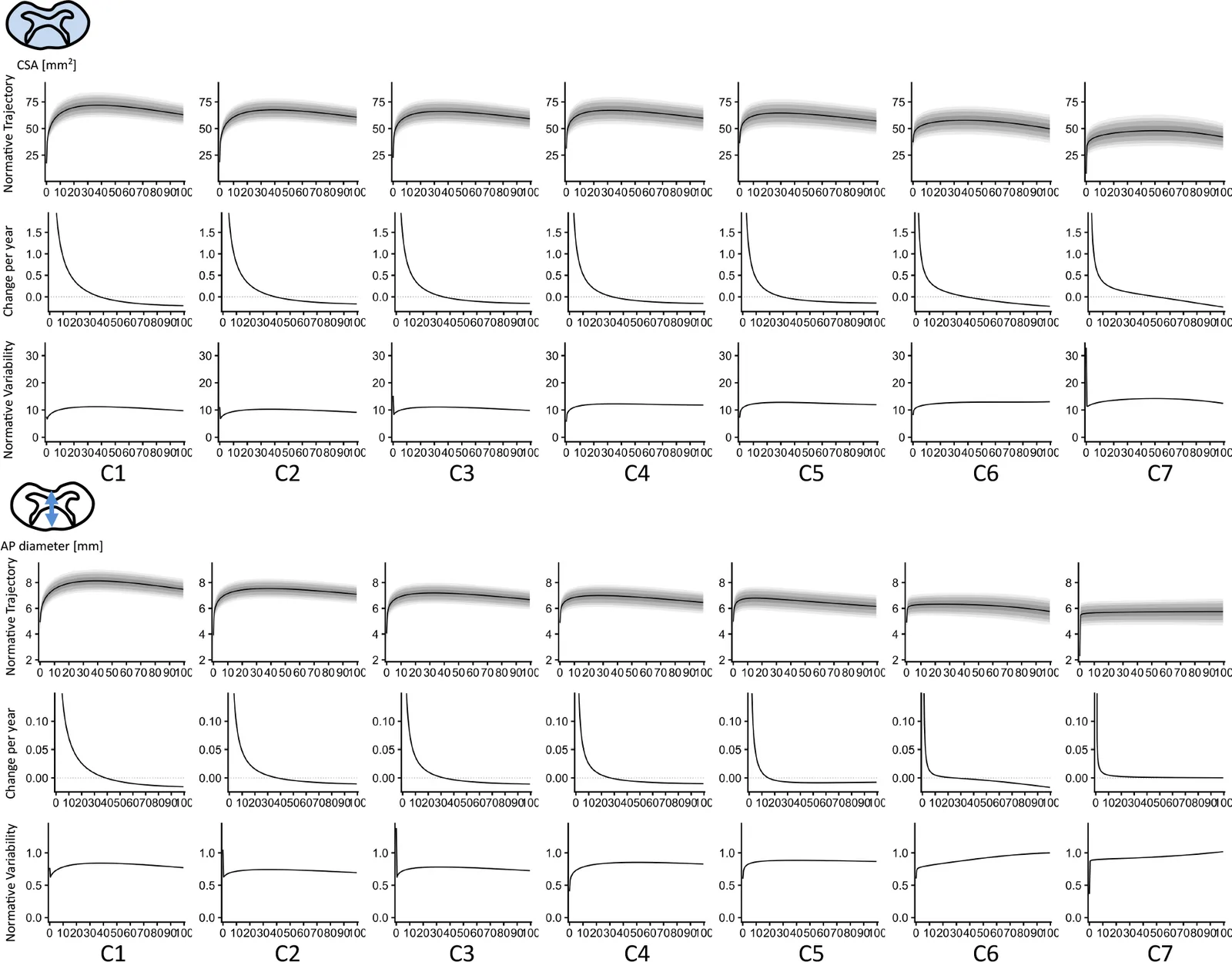
Lifespan Dynamics of Spinal Cord Morphometry Across the Vertebral Levels Figure displays the normative trajectory (centiles), annualized rate of change (change-per-year), and normative population variability (standard deviation, σ) for Cross-Sectional Area (CSA, top) and Anterior-Posterior (AP) Diameter (bottom). Normative trajectories reveal distinct developmental and aging patterns that vary by vertebral level; annualized rate of change quantifies these dynamics, highlighting periods of rapid growth in childhood and adolescence followed by a steady rate of atrophy in late adulthood; Population variability illustrates the spread of measurements changes across the lifespan.

Figure 3 extends these results across C1–C7 for CSA and AP diameter, presenting (i) the normative lifespan trajectory (median), (ii) the annualized rate of change derived from the first derivative, and (iii) population variability (σ). While the global pattern (growth → plateau → decrease) is conserved, the timing and magnitude vary by vertebral level. Parallel panels for RL diameter, compression ratio, eccentricity, and solidity are provided in **Supplementary Figure 2**, and complete centile tables for all features, levels, and sexes are provided as **Supplementary Data**.

### Regional Variations Along the Spinal Cord with Age

**Figure 4.**
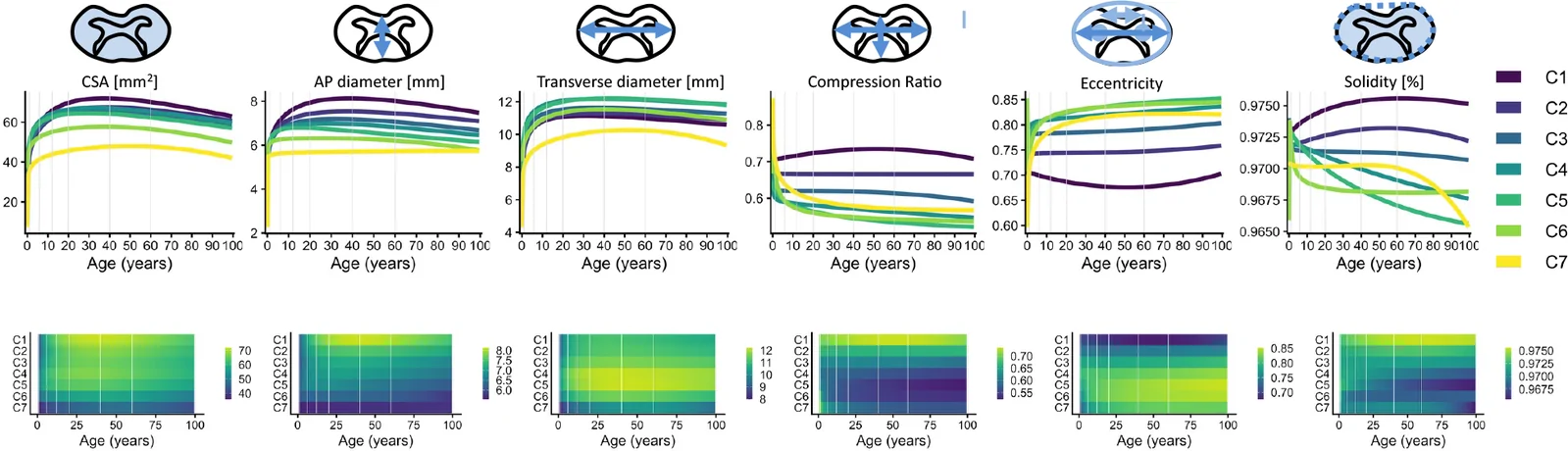
Regional variations along the spinal cord with age. Median lifespan trajectories for all six morphometric features are plotted for each cervical vertebral level (C1–C7) (top row), alongside two-dimensional heatmaps visualizing the magnitude of each feature across both age and vertebral level (bottom row). While overall trends are shared across levels, the timing and magnitude of change are level-dependent, and shape metrics (compression ratio, eccentricity, solidity) display more heterogeneous, region-specific dynamics.

The normative lifespan charts reveal level-dependent patterns across C1-C7 vertebral levels (Figure 4). Size metrics (CSA, AP, RL) generally decrease caudally, with a prominent cervical enlargement at C4–C5 driven chiefly by greater RL (transverse) diameter, consistent with neuroanatomy [18, 21]. While the absolute magnitude of size metrics varies across levels, the overall shape of the lifespan trajectory - early growth and late-life decrease - is largely conserved along the cord. In contrast, measures of cord shape, such as compression ratio and eccentricity, exhibit more complex and level-specific lifespan dynamics, with unique patterns of development and aging at different vertebral locations.

### Lifespan Milestones in Spinal Cord Growth and Aging

**Figure 5.**
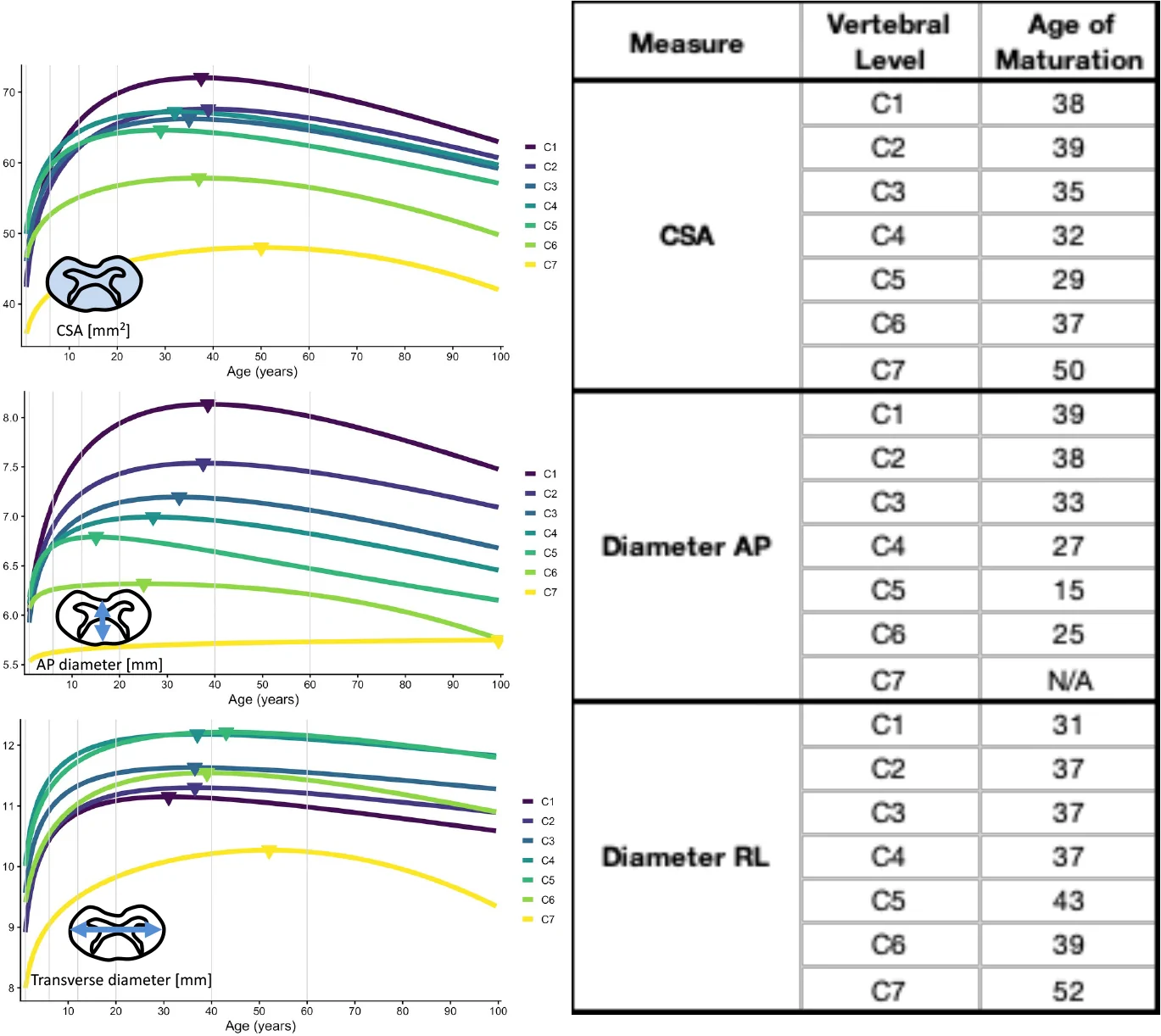
Lifespan milestones in spinal cord growth and maturation. The median lifespan trajectories for Cross-Sectional Area (CSA), Anterior-Posterior (AP) Diameter, and Right-Left (RL) Diameter are shown for each cervical vertebral level. The peak of each curve, interpreted as the age of maturation, is indicated by a marker. The accompanying table quantifies these peak ages for each measure and level. The charts reveal dissociated maturational timelines: CSA consistently peaks in the mid-30s, whereas the AP diameter tends to mature earlier than the RL diameter.

The normative lifespan charts allow for the identification of key developmental milestones, such as the age at which spinal cord morphometric measures reach their peak (Figure 5), which can be interpreted as the age of maturation [23]. CSA peaks in the throughout the 30s across all cervical levels, refining prior estimates from smaller single-site adult cohorts [23](N=129), which suggested a peak around 45 years of age. The maturational timelines for the cord’s diameters are dissociated: the AP diameter tends to peak earlier (~25-40 years), while the RL (transverse) diameter peaks later (~30-50 years) (see Discussion on limitations of C6/C7). Identifying these peaks provides quantitative, non-invasive benchmarks of the transition from developmental growth to the onset of age-related structural decline.

### Quantifying Annualized Rates of Change for Clinical Benchmarking

To establish quantitative benchmarks for clinical and research applications, we derived the annualized rates of change from the normative curves for each morphometric feature across distinct lifespan stages (Table 1). The spinal cord changes most rapidly in early life: in infancy CSA increases dramatically, with early childhood growth of roughly 2-8%/year (metric- and level-dependent). A broad plateau follows, then late adulthood shows a steady but modest decrease: CSA decreases by ~0.2–0.3%/year, and AP/RL diameters by ~0.1–0.2%/year. Absolute rates (e.g., mm^2^/year, mm/year) and stage-wise results for shape metrics are provided in **Supplementary Table 2**.

### Charting Population Variability Across the Lifespan

To characterize normative population variability, we calculated the coefficient of variation (CV) by lifespan stage (Table 2). For size metrics, CVs are largely stable across age: CSA CV is <10% at most levels, with slightly higher values at C6–C7 (likely due to smaller sample sizes and increased susceptibility to algorithmic variance at the edge of the imaging field-of-view) and a modest increase in middle/late adulthood. AP and RL diameters show a similar pattern, maintaining CV <10% across stages. Stage-wise SD and CV for all features and levels are reported in **Supplementary Table 3**.

### Sex Differences in Spinal Cord Morphometry

**Figure 6.**
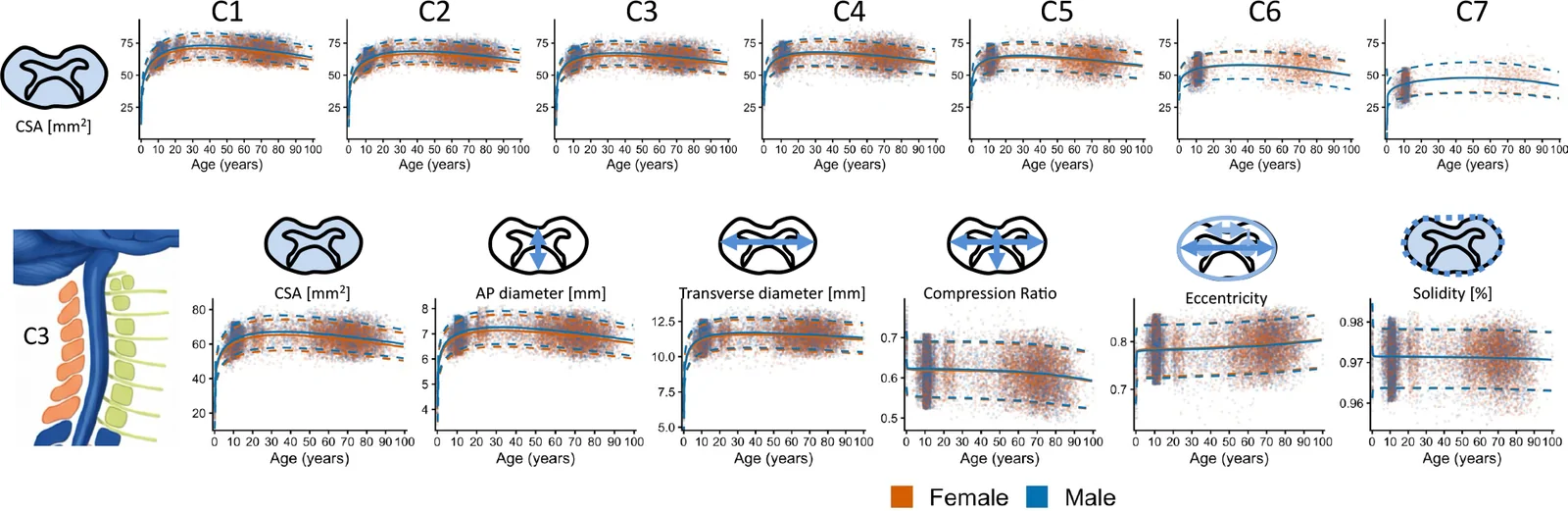
Sex-specific lifespan trajectories of spinal cord morphometry. The top panel displays sex-stratified centile curves (5th, 50th, 95th) for Cross-Sectional Area (CSA) across all seven cervical levels (C1–C7). The bottom panel shows the trajectories for all six morphometric features at the representative C3 level. In all plots, raw data points and fitted centiles are shown for males (blue) and females (red). The charts demonstrate that while males consistently have larger cord size (CSA and diameters) than females, the fundamental non-linear pattern of growth, maturation, and decrease across the lifespan is conserved between sexes.

To characterize sex-specific differences, we modeled lifespan trajectories for males and females by including sex as a fixed-effect covariate for both location and scale (Figure 6). Males have larger CSA and diameters by approximately 2–3% at all cervical levels, whereas the trajectory shapes (growth, maturation, decline) and dispersion/variability are similar between sexes. Shape metrics (compression ratio, eccentricity, solidity) show minimal sex effects. These findings motivate sex-specific centiles for accurate individual benchmarking.

### Brain-Spinal Cord Relationships

**Figure 7.**
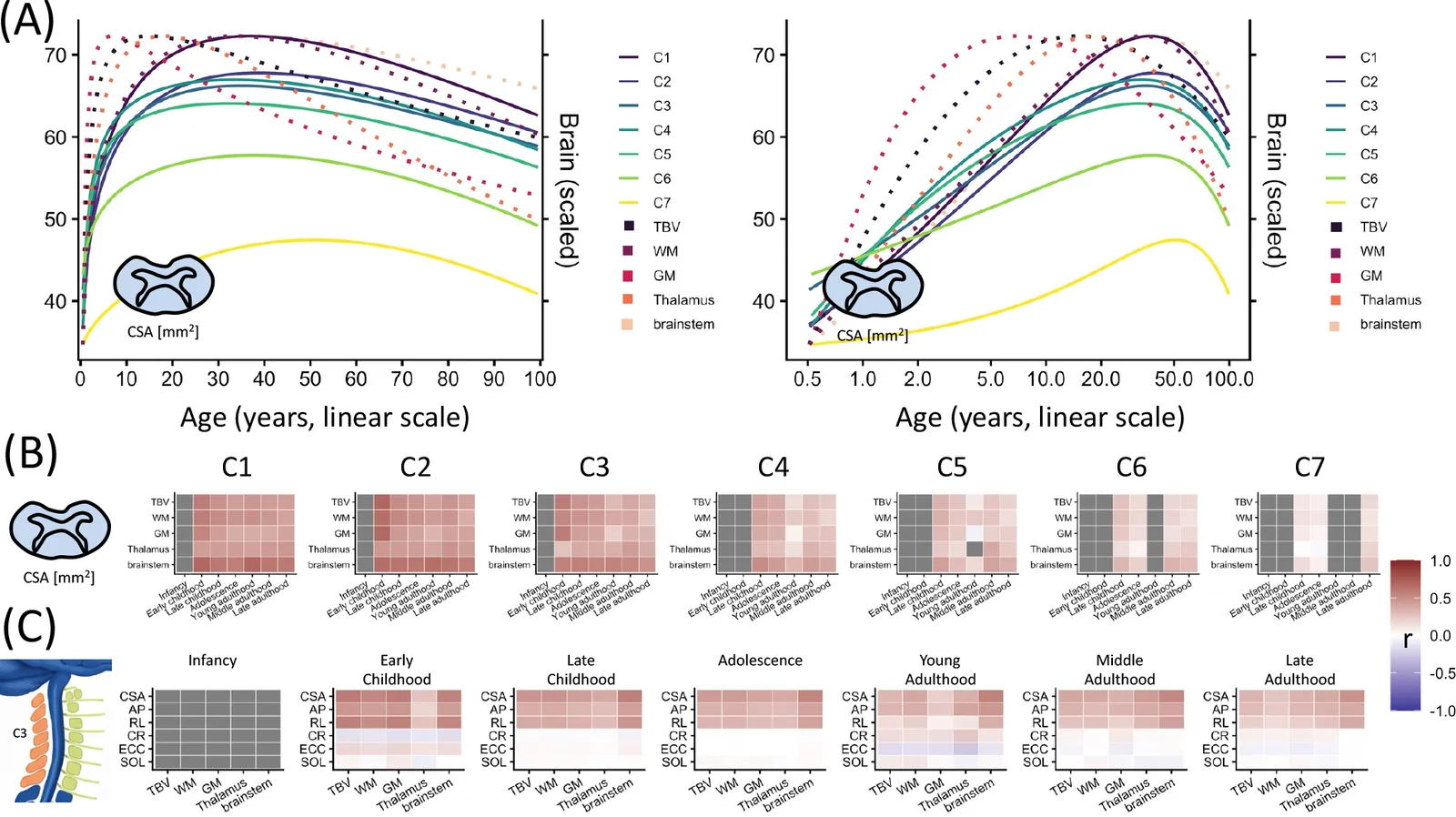
Brain-spinal cord relationships across the lifespan. (A) The median (50th centile) lifespan trajectories for spinal cord Cross-Sectional Area (CSA) at all cervical levels are plotted alongside key brain morphometric volumes. Trajectories are shown on both a linear and a log-scale age axis to accentuate developmental and aging patterns. (B) Pearson correlation coefficients between CSA and brain volumes at different lifespan stages are shown at each cervical level (C1–C7). (C) Correlations between all spinal cord features (at the representative C3 level) and brain volumes are also stratified by lifespan stage. Correlation analyses excluded infancy, as brain volumetric measurements were not consistently reliable in this age range, and any age bin with fewer than 300 subjects. Abbreviations: TBV, total brain volume; WM, total white matter volume; GM, total gray matter volume. We note x-limit is constrained to >0.5 years so that dynamic contrast across the lifespan is visible.

To place spinal cord development and aging within the context of the entire central nervous system, we compared its lifespan trajectory with key brain volumes (Figure 7A). The timing of spinal cord growth, maturation, and aging most closely mirrors the lifespan trajectories of total white matter and brainstem volume, with gray matter, thalamic, and total brain volumes peaking earlier in the lifespan. Lifespan-stage correlations (Figure 7B–C) are positive and strongest in childhood/adolescence, decline from rostral to caudal levels, and are most robust for brainstem (followed by white matter). Shape metrics show little to no association with brain volumes. Correlation analyses excluded infancy where brain volumetry did not meet QC and age-stage cells with insufficient sample size.

## Discussion

We generated vertebral-level, sex-stratified normative charts for cervical spinal cord morphometry by combining 30 population brain cohorts (78,269 scans from 41,042 individuals, ages 0–100, resulting in a total cross-sectional sample of 25,190 healthy individuals) and harmonizing measurements with contrast-agnostic segmentation [2]. These charts model the full distribution of CSA, diameters (AP, RL), and shape indices (compression ratio, eccentricity, solidity) across the lifespan, revealing non-linear trajectories - rapid early growth, a mid-adult peak (mid-30s for CSA), and gradual decrease - with clear level-wise differences and stable, low relative dispersion. Together with sex-stratified curves and brain-cord comparisons, the resource provides a population reference that addresses long-standing inter-individual variability [23 and enables age- and sex-adjusted interpretation of individual measurements.

### Spinal Cord Charts as a Tool for Normative Assessment

Normative reference charts for the spinal cord may potentially be a powerful new tool for clinical and research applications. These charts translate absolute spinal cord measurements into age- and sex-adjusted centile scores, directly paralleling pediatric growth and recent brain charts [31, 44]. A centile expresses where an individual lies within the healthy distribution at their age/sex, clarifying whether a small CSA reflects pathology or the lower tail of typical variation. In clinical contexts, a value below a prespecified centile (e.g., 5th) may flag atypical caliber even when absolute thresholds are ambiguous, improving interpretability for disorders where cord atrophy is relevant.

Clinically, this framing helps in scenarios where variability obscures signal: (i) early disease (e.g., suspected inflammatory or neurodegenerative cord involvement) where a patient’s CSA or diameter is well below age-/sex-expected centiles despite modest absolute deviations; (ii) heterogeneous datasets (different scanners/protocols) where centiles provide a common interpretive scale; and (iii) monitoring where a drop across centiles over time suggests accelerated decline beyond age-expected change. In each case, centiles might complement radiological assessment and absolute metrics, adding standardized context to individual measurements.

For research and trials, centile scores offer variance reduction (age/sex accounted for by design) and standardized endpoints to compare groups or track change relative to normative trajectories. Trial planners can use the stage-specific annualized rates of change to set realistic progression expectations and power calculations, while analyses can test whether interventions preserve centiles (or slow centile decline) versus control. In routine studies, centiles provide a common scale across sites and protocols, supporting reproducible cross-cohort comparisons.

### Using the spinal cord charts

To facilitate practical application, we favored simplicity and accessibility; analogous to pediatric growth charts, we provide reference tables (CSVs) containing sex-stratified centiles at 0.5-year intervals for every morphometric feature and vertebral level. For a new individual, a feature of interest can be derived and instantly and intuitively placed at the nearest age- and sex-adjusted centile. Critically, valid comparison requires that features be derived using the same processing pipeline and version to avoid methodological bias. While not a standalone diagnostic tool, this “centile scoring” enables the visualization of an individual’s development or aging relative to the population, facilitating the identification of trends or extreme deviations (e.g., <5th or >95th percentiles). For more advanced research applications, such as harmonizing new datasets to these norms, we provide the full GAMLSS model objects. This allows for out-of-sample alignment [31, 38], a strategy successfully used in brain charting to overcome scanner or site effects - which we found to be low in our analysis (average <3% across levels C1–C3; **Supplementary Data**).

This resource builds upon previous normative efforts in the literature, representing a shift in scale and scope. Previous work by Frostell et al. [3] provided essential aggregate reviews of segmental diameters (primarily from postmortem examinations), while Papinutto et al. [22, 23, 45] advanced the field by establishing variability and normalization strategies in targeted adult and pediatric cohorts. Most recently, Valošek et al. [21] introduced a high-resolution database of healthy controls (N=307) standardized to the PAM50 template space. Our work complements these efforts by prioritizing sample size and lifespan coverage. Whereas Valošek et al. maximized spatial precision (slice-wise analysis in template space) in a focused cohort, we leveraged vertebral-level aggregation to maximize statistical power across the entire human lifespan (N=25,193; ages 0–100). This trade-off sacrifices some spatial granularity for the robust modeling of age-related variance. Future work may aim to bridge these approaches, potentially mapping our large-scale lifespan variance into the PAM50 coordinate system to combine high spatial fidelity with robust, data-driven lifespan norms.

Crucially, while these charts define population-level expectations, establishing the functional relevance of individual deviations remains a priority for future validation. Emerging evidence links cervical atrophy directly to clinical outcomes, including respiratory dysfunction in ALS [8], cognitive decline in Alzheimer’s disease [9], and irreversible motor disability in MS [7] - suggesting that ‘centile scoring’ may eventually serve as a predictive biomarker for functional impairment.

### Biological Insights into Lifespan Dynamics of the Spinal Cord

Our lifespan charts provide insights into the biological processes underlying spinal cord development, maturation, and aging. By quantitatively mapping the development trajectory, they revealed rapid growth in early life followed by a protracted maturation extending well into early adulthood [34]. We observe that CSA and cord diameters continue to increase through adolescence, reaching peak values around the mid-30s for CSA. This timeline suggests that the structural maturation of the human spinal cord persists considerably longer than puberty, likely driven by ongoing myelination and axonal growth during young adulthood. Indeed, extensive literature demonstrates that myelination within the central nervous system, in all association, projection, and commissural pathways, continues into the third and even fourth decades of life [46–52]. Our finding of a peak CSA in the mid-30s refines previous estimates from smaller adult cohorts which suggested a peak slightly later, around 45 years [23], potentially reflecting the enhanced temporal resolution afforded by our large-scale dataset.

Following this peak, our charts also quantify the gradual decreases in cord morphometry in aging. While age effects are modest through middle age, a measurable atrophy becomes evident in middle/late adulthood, accelerating somewhat in the oldest individuals. The underlying neurobiology of this late-life cord atrophy likely involves a multifactorial process analogous to brain aging, including progressive demyelination, axonal loss, and neuronal shrinkage or death in spinal gray matter. This reinforces the view of the spinal cord as an integral part of the CNS aging process and suggests its metrics can act as a complement to brain measures. In summary, our charts delineate key biological milestones - the age of peak maturation and the onset and rate of age-related decline - providing a quantitative framework for understanding spinal cord structure across the lifespan.

### An Integrated Central Nervous System: Brain–Cord Relationships and Sex Differences

Our findings reinforce the view of the central nervous system developing and aging as an integrated unit [53, 54], highlighting both coordinated brain-cord dynamics and sex differences. We find strong positive cross-sectional correlations between cervical cord CSA and global brain volumes (total brain, white matter, gray matter, thalamus, brainstem)[22, 25, 55], particularly during developmental periods (Figure 7B, C). This brain–cord coupling suggests an intuitive scaling effect: individuals with larger brains tend to have proportionally larger spinal cords, likely reflecting shared genetic and developmental influences on overall nervous system size [25]. This relationship motivates and highlights the rationale behind normalization strategies that adjust spinal cord measures for intracranial or brain volume to reduce inter-subject variability [1, 5, 12, 22, 23] – unfortunately, brain images are not ubiquitously available for all spinal cord studies. Further, the lifespan trajectories show spinal cord morphometry changes closely paralleling those of brain white matter and brainstem volume (Figure 7A). This indicates that the spinal cord does not age in isolation but rather undergoes senescence in concert with the brain, again suggesting shared underlying biological mechanisms [14]. In pathological states like MS, this link is often more pronounced, with concurrent brain and spinal cord atrophy reflecting widespread neurodegeneration [15, 56, 57]. Understanding this normative brain-cord relationship opens possibilities for developing combined brain-plus-cord centile scores as more holistic biomarkers of CNS health, aging, or disease burden, potentially identifying individuals where one component deviates unexpectedly relative to the other.

We also confirmed sex differences in spinal cord morphometry, necessitating the use of sex-stratified normative charts (Figure 6). Males exhibited consistently larger cervical CSA and diameters than females across the lifespan. This sexual dimorphism likely reflects known differences in overall body size, spinal canal dimensions, and potentially hormonal influences on neural development. While the absolute scale differs, the qualitative shape of the lifespan trajectory and patterns of population variability are similar between sexes. Practically, this means that comparing an individual’s measurement to sex-specific norms is necessary for accurate assessment and avoids misclassification due to inherent size differences. Our sex-stratified charts provide this calibration, ensuring appropriate benchmarking for both males and females throughout life.

### Limitations and Future Directions

While these first lifespan charts represent a significant advance for spinal cord morphometry, several limitations should be acknowledged. First, our models are derived from cross-sectional data, which approximates lifespan trajectories but cannot capture individual longitudinal changes or potential cohort effects [58]. Although our approach mirrors recent large-scale brain charting initiatives [31], future integration of longitudinal data is needed to fully validate these normative curves. However, our stability analyses provide confidence in the current models: bootstrapping cross-sectional samples yielded remarkably low uncertainty (CV < 0.2% for most features/ages), and a leave-one-site-out analysis confirmed robustness to the influence of individual contributing studies (CV < 1% for C1-C5), suggesting stable population estimates despite the cross-sectional design (**Supplementary Figures 3–8**).

Further limitations include the uneven sampling across age groups and lower sample sizes at caudal levels C6 and C7 (Figure 1), which reduces model precision at the extremes of the age range and lower cervical spine. Moreover, because the cervical cord lies at the periphery of the field of view in standard brain MRI protocols, measurements at these caudal levels are susceptible to gradient non-linearity distortions, a known source of morphometric variability that is particularly pronounced at the edges of the scanner field. This technical confound likely contributes to the larger coefficient of variation observed at C7 compared to rostral levels, rendering maturation and peak estimates less precise in this region. Regarding the age distribution, infancy (<1 years old) show large coefficients of variation, which explains the large % change per year (Table 1) and population variability (Table 2), We advocate caution in the use of these charts in this age range, as only two datasets with few samples included this developmental period. Fitting with a log-age range was tested, with similar results. Future work should include a more dense sampling of early and late-infancy.

Methodologically, our charts are anchored to *vertebral* levels, a practical MRI landmark, rather than functional *neuronal* segments. This is particularly relevant for lifespan modeling because the spine and spinal cord do not grow at the same rate, causing the spatial correspondence between vertebral levels and underlying spinal segments to shift with age. Because of this, observed age-related changes in morphometry at a fixed vertebral level could partially reflect this shifting anatomical frame of reference rather than solely intrinsic tissue changes. While vertebral referencing aligns with common clinical practice, and is practically feasible with noninvasive clinical MRI, future work could map these norms onto segmental anatomy [3, 59] or use developmentally stable landmarks like the pontomedullary junction [1]. Next, our charts are restricted to cervical cord due to the nature of repurposed brain imaging datasets; future work can extend charting to the thoracic and lumbar cord using dedicated spine datasets. The normative values are also inherently tied to the state-of-the-art contrast-agnostic segmentation method employed; application to data processed with different algorithms may require calibration and/or harmonization. Next, averaging across vertebral levels versus slice-wise (e.g. in PAM50 space, as in [21]) may overshadow more subtle changes happening within a level or at disc levels (i.e., stenosis). Moreover, we covary for age and sex, but other factors could explain variance – ethnicity, size, neck length, etc, and it remains to be seen how best to integrate these phenotypes to identify typical versus atypical spinal cord. Finally, while site effects were statistically modeled, residual variance due to scanner hardware or specific protocols might persist [26, 27]. The crucial next step is validating the clinical utility of these charts by applying centile scoring in patient populations (e.g., MS, SCI, DCM) to determine their sensitivity in detecting atypical development or pathological atrophy. Integrating these spinal cord charts with existing brain charts could also enable powerful, composite biomarkers for assessing overall CNS health and disease.

## Conclusion

By using large-scale brain imaging cohorts with a contrast-agnostic segmentation approach, we have generated the first comprehensive normative charts detailing the non-linear dynamics of cervical spinal cord morphometry across the human lifespan. These charts provide reference data on typical growth, maturation, aging, sex differences, and population variability, revealing parallel development with brain structures like white matter and the brainstem. Analogous to established growth charts, these spinal cord charts offer a framework for converting individual measurements into age- and sex-adjusted centile scores, and a standardized tool to benchmark spinal cord health and detect atypical variations indicative of neurodevelopmental or neurodegenerative processes. This work lays the foundation for more precise, individualized assessment of spinal cord integrity in clinical practice and research, with the potential to enhance diagnostic accuracy, improve statistical power in clinical trials, and deepen our understanding of central nervous system development and aging. Future extensions incorporating longitudinal data, thoracic/lumbar regions, and clinical populations will further refine these essential neuroanatomical resources. The lifespan charts, model fits, and out-of-sample alignment code have been made available at (10.5281/zenodo.18306654) to facilitate the use and application of these charts.

## Figures and Tables

**Table 1.** Annualized Percentage Rates of Change for Spinal Cord Size Metrics.

measure	Vetebral Level	Infancy	Early Childhood	Late Childhood	Adolescence	Young Adulthood	Middle Adulthood	Late Adulthood
**CSA**	**C1**	150	7.6	1.8	0.75	0.16	−0.13	−0.27
	**C2**	140	6.7	1.6	0.68	0.16	−0.093	−0.22
	**C3**	110	5.4	1.3	0.51	0.09	−0.11	−0.22
	**C4**	69	4.6	1	0.4	0.044	−0.13	−0.22
	**C5**	45	3.9	0.87	0.32	0.0072	−0.14	−0.22
	**C6**	31	2.8	0.79	0.38	0.095	−0.11	−0.32
	**C7**	400	3.4	0.97	0.52	0.23	0.0017	−0.32
**Diameter AP**	**C1**	27	3.8	1.2	0.52	0.12	−0.074	−0.17
	**C2**	61	3.5	0.83	0.34	0.074	−0.057	−0.13
	**C3**	56	3	0.68	0.26	0.031	−0.082	−0.14
	**C4**	32	2.2	0.47	0.16	−0.012	−0.097	−0.14
	**C5**	30	2	0.24	−0.0058	−0.1	−0.13	−0.13
	**C6**	32	0.82	0.11	0.034	−0.013	−0.07	−0.19
	**C7**	170	0.4	0.1	0.055	0.029	0.016	0.0076
**Diameter RL**	**C1**	80	3.8	0.72	0.24	0.02	−0.064	−0.093
	**C2**	81	3.8	0.77	0.28	0.052	−0.039	−0.072
	**C3**	67	3.2	0.63	0.23	0.042	−0.033	−0.06
	**C4**	53	3.1	0.63	0.23	0.044	−0.031	−0.058
	**C5**	31	2.8	0.69	0.3	0.085	−0.021	−0.076
	**C6**	15	2.5	0.79	0.36	0.088	−0.049	−0.12
	**C7**	100	2.9	0.82	0.44	0.2	1.70E-02	−0.23
**Compression Ratio**	**C1**	0.16	0.15	0.13	0.11	0.068	0.0045	−0.091
	**C2**	−2.4	−0.14	−0.011	−0.0037	−0.0012	−0.00042	−0.00018
	**C3**	−5.2	−0.069	−0.012	−0.0083	−0.015	−0.036	−0.091
	**C4**	−27	−0.55	−0.14	−0.097	−0.081	−0.075	−0.073
	**C5**	−5	−1.1	−0.46	−0.29	−0.17	−0.1	−0.062
	**C6**	12	−3.7	−0.62	−0.26	−0.11	−0.05	−0.027
	**C7**	−26	−2.5	−0.68	−0.34	−0.16	−0.066	−0.019
**Eccentricity**	**C1**	−0.31	−0.17	−0.14	−0.12	−0.071	−0.0047	0.095
	**C2**	8	0.098	0.014	0.0067	0.0073	0.015	0.037
	**C3**	6.4	0.079	0.015	0.012	0.017	0.026	0.041
	**C4**	19	0.35	0.074	0.048	0.038	0.035	0.033
	**C5**	2.8	0.8	0.24	0.13	0.073	0.043	0.027
	**C6**	−25	2	0.35	0.15	0.061	0.028	0.014
	**C7**	25	1.7	0.45	0.22	0.091	0.029	−3.30E-03
**Solidity**	**C1**	0.027	0.015	0.0097	0.0067	0.0037	1.10E-03	−1.20E-03
	**C2**	−0.013	0.0053	0.0047	0.004	0.0025	4.70E-04	−0.0026
	**C3**	−0.36	−0.0048	−0.00067	−0.00031	−0.0003	−0.00057	−1.40E-03
	**C4**	0.3	0.0037	−0.006	−0.0061	−0.0054	−0.0046	−0.0038
	**C5**	−0.05	−0.023	−0.016	−0.012	−0.009	−0.0062	−0.0038
	**C6**	1.1	−0.072	−0.01	−0.004	−0.0015	−0.00044	0.00019
	**C7**	−0.024	−0.0033	−0.00082	8.10E-05	6.10E-04	−0.0012	−0.012

**Table 2.** Population Variability of Spinal Cord Size Metrics Across the Lifespan.

		Coefficient of Variation						
measure	Vetebral Level	Infancy	Early Childhood	Late Childhood	Adolescence	Young Adulthood	Middle Adulthood	Late Adulthood
**CSA**	**C1**	15	8.2	7.9	7.9	7.8	7.8	7.7
	**C2**	20	8.1	7.8	7.7	7.6	7.6	7.6
	**C3**	22	8.9	8.6	8.5	8.4	8.4	8.3
	**C4**	9.1	9.1	9.1	9.1	9.1	9.2	9.5
	**C5**	9.9	9.9	9.9	9.9	9.9	10	10
	**C6**	11	11	11	11	11	11	12
	**C7**	120	15	15	15	15	15	15
**Diameter AP**	**C1**	6.6	5.3	5.2	5.2	5.2	5.2	5.2
	**C2**	9.5	5.2	5	5	4.9	4.9	4.9
	**C3**	11	5.5	5.4	5.4	5.4	5.4	5.4
	**C4**	4.5	5.4	5.7	5.8	6	6.2	6.3
	**C5**	6.1	6.2	6.3	6.4	6.6	6.7	6.9
	**C6**	6.2	6.3	6.4	6.5	6.8	7.3	8.1
	**C7**	8	8	8	8	8.1	8.2	8.5
**Diameter RL**	**C1**	8.5	5	4.9	4.8	4.8	4.8	4.8
	**C2**	11	5.3	5.1	5	5	5	4.9
	**C3**	12	5.8	5.6	5.6	5.5	5.5	5.5
	**C4**	5.9	5.9	5.9	5.9	6	6.1	6.3
	**C5**	6.2	6.2	6.2	6.2	6.3	6.3	6.6
	**C6**	6.6	6.6	6.6	6.6	6.6	6.7	7
	**C7**	50	9.8	8.7	8.4	8.2	8	8

## Data Availability

The lifespan charts, model fits, and out-of-sample alignment code have been made available at (10.5281/zenodo.18306654) to facilitate the use and application of these charts. Data used in the preparation of this article were obtained from the Alzheimer’s Disease Neuroimaging Initiative (ADNI) database (adni.loni.usc.edu). The ADNI was launched in 2003 as a public-private partnership, led by Principal Investigator Michael W. Weiner, MD. The primary goal of ADNI has been to test whether serial magnetic resonance imaging (MRI), positron emission tomography (PET), other biological markers, and clinical and neuropsychological assessment can be combined to measure the progression of mild cognitive impairment (MCI) and early Alzheimer’s disease (AD). Data from the Alzheimer’s Disease Neuroimaging Initiative (ADNI) are available upon request from https://adni.loni.usc.edu/. Data from the AOMIC-PIOP1 dataset are freely available for download on OpenNeuro: https://openneuro.org/datasets/ds002785/versions/2.0.0. Data from the AOMIC-PIOP2 dataset are freely available for download on OpenNeuro: https://openneuro.org/datasets/ds002790/versions/2.0.0. Data from the AOMIC-ID1000 dataset are freely available for download on OpenNeuro: https://openneuro.org/datasets/ds003097/versions/1.2.1. Data from the Boston Adolescent Neuroimaging of Depression and Anxiety (BANDA) dataset are available upon request from https://www.humanconnectome.org/study/connectomes-related-anxiety-depression. Data from BIOCARD are available upon request after filling out a data use application: https://www.gaaindata.org/partner/BIOCARD. Data from the Baltimore Longitudinal Study of Aging (BLSA) are available upon request from https://www.blsa.nih.gov/. Data from the Centre for Attention Learning and Memory (CALM) dataset are available upon request from https://calm.mrc-cbu.cam.ac.uk/researchers/. Data from the Cambridge Center for Ageing Neuroscience (CAMCAN) dataset are available upon request from https://camcan-archive.mrc-cbu.cam.ac.uk/dataaccess/. Data from the Dallas Lifespan Brain Study (DLBS) dataset are freely available for download on OpenNeuro: https://openneuro.org/datasets/ds004856/versions/1.2.0. Data from the Health & Aging Brain Study - Health Disparities (HABS-HD) dataset are available upon request from https://apps.unthsc.edu/itr/reports. Imaging data and basic demographic information for the Healthy Brain Network (HBN) dataset are freely available to download from https://fcon_1000.projects.nitrc.org/indi/cmi_healthy_brain_network/. Phenotypic data are available upon request by filling out a data use agreement: https://fcon_1000.projects.nitrc.org/indi/cmi_healthy_brain_network/Phenotypic.html. Data from the Human Connectome Project – Aging (HCPA) dataset are available upon request from https://www.humanconnectome.org/study/hcp-lifespan-aging. Data from the International Consortium for Brain Mapping (ICBM) dataset are available upon request from www.loni.usc.edu/ICBM. Data from the Memory and Aging Project (MAP), Religious Orders Study (ROS), and the Minority Aging Research Study (MARS) datasets are available upon request from https://www.radc.rush.edu/. Data from the Multisite, Multiscanner, and Multisubject Acquisitions for Studying Variability in Diffusion Weighted Magnetic Resonance Imaging (MASiVar) dataset are freely available for download on OpenNeuro: https://openneuro.org/datasets/ds003416/versions/2.0.2. Data from the National Alzheimer’s Coordinating Center (NACC) and the Standardized Centralized Alzheimer’s & Related Dementias Neuroimaging (SCAN) are available upon request from https://naccdata.org/requesting-data/data-request-process. Data from the Pediatric Imaging, Neurocognition, and Genetics dataset (PING) are available for download from the National Institutes of Mental Health data archive upon request from https://nda.nih.gov/. Data from the UCLA Consortium for Neuropsychiatric Phenomics LA5c Study (UCLA) dataset are freely available for download on OpenNeuro: https://openneuro.org/datasets/ds000030/versions/1.0.0. Data from the Vanderbilt Memory and Aging Project (VMAP_JEFFERSON, VMAP_2.0) are available upon request from https://vmacdata.org/vmap/data-requests. Data from the Vanderbilt Alzheimer’s Disease Research Centre’s Tennessee Alzheimer’s Project (TAP) are available from https://vmacdata.org/vadrc-tap. Data from the Wisconsin Registry for Alzheimer’s Prevention (WRAP) are available upon request from https://wrap.wisc.edu/data-requests-2/. Data from the Adolescent Brain Cognitive Development (ABCD) Study are available for download upon request from (https://abcdstudy.org/scientists/data-sharing/). Data from the Bipolar & Schizophrenia Consortium for Parsing Intermediate Phenotypes dataset (BSNIP1) and its renewal (BSNIP2) are available for download from the National Institutes of Mental Health data archive upon request from https://nda.nih.gov/. Vanderbilt University data (MORGAN, BABIES-ABC, CUTTING, VUMC-ASD) subject to third party restrictions. Please contact corresponding author for data requests.
